# A Comparative Analysis in the Treatment of Full-Thickness Wounds: Negative-Pressure Wound Therapy (NPWT) Combined With High-Purity Type I Collagen-Based Skin Substitute Versus NPWT Alone

**DOI:** 10.7759/cureus.96977

**Published:** 2025-11-16

**Authors:** Naveen Narayan, Divakara Raghupathi, Vikram Ramamurthy, Shivannaiah Chethan, Suhas Gowda

**Affiliations:** 1 Plastic Reconstructive and Aesthetic Surgery, Adichunchanagiri Institute of Medical Sciences, B G Nagara, IND; 2 General Surgery, Adichunchanagiri Institute of Medical Sciences, B G Nagara, IND; 3 General Surgery, Shridevi Institute of Medical Sciences and Research Hospital, Tumkuru, IND

**Keywords:** burn wound, chronic leg ulcer, collagen skin substitute, dfu (diabetic foot ulcer), full-thickness wounds, helicoll®, negative-pressure wound therapy, randomized controlled trial, traumatic wound, wound healing

## Abstract

Background: Full-thickness wounds are a significant clinical burden, especially in patients with chronic comorbidities. They pose a major clinical challenge due to their prolonged healing times and high risk of complications. Advanced wound care strategies like negative-pressure wound therapy (NPWT), which enhances wound healing by reducing edema, promoting granulation tissue formation, and removing exudates and bioengineered skin substitutes such as high-purity type I collagen-based skin substitute (HPTC/Helicoll^®^) that acts as an extracellular matrix scaffold to stimulate angiogenesis and cellular proliferation, have emerged as promising interventions. This study evaluates the comparative effectiveness of NPWT combined with HPTC versus NPWT alone in promoting wound healing in full-thickness wounds.

Methods: This was a prospective, randomized, open-label, parallel-group clinical trial conducted at the Department of Plastic, Reconstructive, and Aesthetic Surgery, Adichunchanagiri Institute of Medical Sciences (AIMS), Karnataka, India. This study enrolled 104 patients with full-thickness wounds, randomly allocated into two groups: Group A received NPWT combined with HPTC (n = 52), and Group B received NPWT alone (n = 52). The primary outcome was percentage wound area reduction at seven weeks, while secondary outcomes included time to complete epithelialization, proportion achieving complete closure, vascularity infiltration on histology, pain assessment using the Visual Analog Scale (VAS), quality of life (QoL) outcome using the EuroQol 5-Dimension 5-Level (EQ-5D-5L) questionnaire, and scar assessment using Vancouver Scar Scale (VSS) scores. Statistical analysis included Student’s t-test, Chi-square test, and Kaplan-Meier survival analysis.

Results: Group A showed significantly higher mean wound size reduction (p < 0.01) at seven weeks, with Group A demonstrating a reduction of 89.35% ± 16.08 and Group B showing 57.85% ± 12.73 reduction, with p value <0.001 (highly significant). Complete healing was achieved in 45 patients (86.54%) of Group A compared to 22 patients (42.31%) of Group B by seven weeks, being statistically highly significant (p-value < 0.001). Mean time to wound closure was shorter in Group A (36.81 ± 12.88 days) than in Group B (43.94 ± 16.70 days), showing a statistically superior closure rate. Pain scores on the VAS, QoL Assessment using EQ-5D-5L, and scar assessment using the VSS were also significantly in favor of the combination group compared to the NPWT-alone group.

Conclusion: The combination of NPWT with HPTC skin substitute (Helicoll^®^) significantly accelerates wound healing and faster closure, improves histopathological parameters, and has better scar outcomes in full-thickness wounds compared to NPWT alone. These findings support incorporating HPTC-based skin substitutes in complex wound care protocols, and this combination therapy represents a promising advancement in wound management.

## Introduction

Full-thickness wounds, which extend through the dermis into the underlying subcutaneous tissue or deeper structures, remain a formidable challenge in reconstructive and wound care practice. These wounds may arise from trauma, surgical dehiscence, pressure injuries, or chronic ulceration and are often complicated by infection, vascular insufficiency, and comorbidities such as diabetes and peripheral vascular disease. They affect millions of patients worldwide and impose a substantial economic burden on healthcare systems [1,2]. They are a growing concern in healthcare due to their prolonged healing time, high recurrence, and associated morbidity [3,4]. These wounds often demonstrate delayed healing and increased risk of complications, including infection, chronic inflammation, and impaired tissue regeneration [5,6]. Without optimal management, these wounds fail to heal through the conventional stages of wound repair, functional impairment, increased risk of limb loss, poor cosmetic results, and high healthcare costs. The complex pathophysiology of wound healing involves intricate interactions between cellular components, growth factors, extracellular matrix (ECM) proteins, and vascular elements, necessitating comprehensive therapeutic approaches to optimize clinical outcomes [7].

Negative-pressure wound therapy (NPWT) has emerged as an effective modality to accelerate wound healing by removing excess exudate, enhancing perfusion, promoting granulation tissue formation, and maintaining an optimal wound environment and thus is a cornerstone in the treatment of complex wounds [8,9,10]. By applying controlled sub-atmospheric pressure, NPWT reduces interstitial edema, increases local blood flow, removes exudate, and stimulates granulation tissue formation. The application of controlled negative pressure creates a conducive environment for cellular proliferation and angiogenesis, facilitating the wound healing cascade [11,12]. Despite its advantages, NPWT alone may not suffice for extensive tissue regeneration and achieve rapid epithelialization, especially in large or complex wounds where the ECM and vascular supply have been significantly compromised [13].

The integration of biological skin substitutes in wound healing has emerged as a promising adjunctive therapy to enhance wound healing outcomes [14,15]. High-purity type I collagen (HPTC) skin substitutes, such as Helicoll®, provide a bioactive scaffold that mimics native ECM, facilitating cellular migration, angiogenesis, keratinocyte proliferation, and growth factor delivery. Helicoll® is an acellular dermal replacement product made of high-purity (>97%) type I collagen that provides a framework promoting blood vessel regeneration and biologic cell migration [16]. It is derived from bovine sources but processed to remove antigenic components. Helicoll® undergoes a patented treatment process for purification, making it the least immunogenic and highly biocompatible [17].

These bioengineered constructs offer several advantages, including biocompatibility, biodegradability, and optimal bio-integration with host tissue, while promoting natural healing processes [18,19]. Recent randomized controlled trials (RCTs) have established its efficacy in chronic wound healing, including diabetic foot ulcers (DFUs) and venous leg ulcers (VLUs) [16,20,21]. Narayan et al. demonstrated that HPTC achieved significantly greater wound closure compared to dehydrated human amniot/chorion membrane (dHACM) in DFUs [16]. Similarly, an RCT in VLUs showed faster healing and better granulation quality with HPTC than dHACM [20]. Another dual-country RCT involving 55 patients reported consistent superiority of HPTC across different patient populations and healthcare settings [22]. These studies have shown superior healing rates, reduced healing time, and improved tissue quality compared to standard care approaches.

While these studies establish the role of HPTC in chronic ulcer management, the potential synergistic effect of combining HPTC with NPWT in full-thickness wounds has not been extensively studied. The rationale for combining NPWT with high-purity type-I collagen-based skin substitutes lies in their complementary mechanisms of action. Theoretically, NPWT could optimize the wound bed and vascular environment (mechanical environment), while HPTC provides the structural and biochemical support (biological framework) for accelerated tissue regeneration. This combination may result in faster and more complete healing than NPWT alone, addressing both the macroscopic and microscopic aspects of wound healing, potentially leading to superior clinical outcomes [12,13,20,21,23].

The present study aims to compare the clinical outcomes of NPWT combined with HPTC skin substitute versus NPWT alone in patients with full-thickness wounds, using a randomized controlled design, to provide robust evidence for clinical decision-making in advanced wound care. Our hypothesis is that the combination therapy will result in significantly greater wound area reduction, faster closure, improved scar quality, and better patient-reported pain outcomes, without increasing adverse events.

## Materials and methods

This was a prospective, randomized, open-label, parallel-group clinical trial conducted at the Department of Plastic, Reconstructive, and Aesthetic Surgery, Adichunchanagiri Institute of Medical Sciences (AIMS), Karnataka, India. The study compared the efficacy of NPWT combined with HPTC skin substitute versus NPWT alone in the management of full-thickness wounds. Patient recruitment occurred between March 2025 and August 2025. The anticipated follow-up continued until August 2025, with each participant observed for a total of seven weeks (six weeks of intervention plus one week of follow-up). The trial was registered prospectively with ClinicalTrials.gov (ID: NCT06873867) and approved by the Institutional Ethics Committee (Approval No.: AIMS/IEC/013/2025). The study adhered to the principles of the Declaration of Helsinki and Good Clinical Practice guidelines. All participants provided written informed consent prior to enrollment after a comprehensive explanation of study procedures, risks, and benefits. Patient confidentiality was maintained throughout the study period.

Patients aged 18-80 years with full-thickness wounds (wound depth extending through dermis into subcutaneous tissue), including traumatic, post-surgical dehiscence, burns ulcers, pressure ulcers, DFUs, or VLUs, wound size >20 cm^2^ and <75 cm^2^, with adequate vascular supply to the wound site, ability to comply with treatment protocol and follow-up visits, and provided written informed consent were eligible.

Exclusion criteria included active infection or osteomyelitis requiring systemic antibiotics, active malignancy at the wound site, immunosuppression, uncontrolled diabetes (HbA1c > 9%), on systemic corticosteroids, patients on anticoagulation therapy with INR >3.0 used advanced wound care products within the last 30 days, autoimmune or connective tissue disorders, or allergy to bovine collagen.

Sample size

Sample size calculation was based on the primary outcome of wound area reduction. Assuming a mean difference of 30% in wound area reduction between groups, with a standard deviation of 20%, power of 80%, and alpha of 0.05, a minimum of 47 patients per group was required. Accounting for a 10% dropout rate, at 52 patients per group, with a total of 104 patients were recruited. This calculation was informed by effect sizes reported in previous HPTC RCTs in diabetic foot, venous leg, and pressure ulcers, which demonstrated mean differences of 10-15% in wound healing outcomes [6,7].

Randomization and blinding

Participants were randomized 1:1 into two treatment groups using a computer-generated randomization sequence with block sizes of 4, 6, and 8. Patients were assigned to either Group A (NPWT + HPTC) or Group B (NPWT alone). Randomization was stratified by wound etiology. Allocation concealment was maintained using sequentially numbered, opaque, sealed envelopes prepared by a research assistant not involved in patient care or outcome assessment. Due to the nature of the intervention, complete blinding was not feasible; however, outcome assessors, pathologists, and data analysts were blinded to treatment allocation.

Intervention protocol

Group A (NPWT + HPTC)

The wound was first debrided to remove necrotic tissue and ensure a viable wound bed. A sheet of HPTC skin substitute (Helicoll®) was applied to completely cover the wound surface. This was followed by a non-adherent porous dressing layer. NPWT was then applied using polyurethane foam at ~125 mmHg intermittent pressure (10 minutes on, two minutes off) for five to seven days. The dressing was changed as per SOC thereafter, with repeat application of HPTC and NPWT as needed.

Group B (NPWT Alone)

Following debridement, a non-adherent porous dressing was applied directly over the wound. NPWT was then initiated with the same parameters (polyurethane foam, ~125 mmHg, intermittent mode) and followed by SOC dressing. NPWT was reapplied if required.

All patients received standardized wound care, including sharp debridement as needed to remove non-viable tissue, infection control with topical antimicrobials when indicated, pressure redistribution and offloading as appropriate, nutritional assessment and optimization, glycemic control for diabetic patients, compression therapy for venous ulcers, and pressure offloading for wounds when appropriate. Patient education on wound care was given. Repeat application of NPWT + HPTC or NPWT alone was considered based on clinical assessment of wound-bed readiness, specifically the presence of healthy granulation tissue, reduction of slough or necrotic tissue, and absence of active infection.

Outcome measures

The primary outcome of the study was the percentage wound area reduction from week 1 through week 6, plus one week of follow-up, measured using standardized digital photography with calibrated scaling to ensure consistent and objective documentation. Measurements were performed by trained personnel blinded to treatment allocation.

Secondary outcomes included time to complete wound closure (in days) within seven weeks, proportion of patients achieving complete closure by week 7, histological vascularity infiltration, between baseline (Day 0) and Day 5 biopsies, mean number of applications of the skin substitute required, incidence of adverse events (infection, allergic reaction, bleeding, maceration), pain score change using a 0-10 Visual Analog Scale (VAS) [24] from baseline to week 6, quality of life (QoL) assessment using the EuroQol 5-Dimension 5-Level (EQ-5D-5L) [25], and scar quality assessed at week 7 using the Vancouver Scar Scale (VSS) [26].

Complete wound closure is defined as 100% epithelialization. Histopathological assessment included taking punch biopsies (2 mm) from the wound edge at baseline and day 5 post-intervention under local anesthesia (2% lidocaine without epinephrine). Fixation in 10% neutral buffered formalin for 24 hours, processing through graded alcohol and paraffin embedding, and serial sectioning at 4 μm thickness were done. Samples were processed using standard histological techniques and stained with hematoxylin and eosin (H&E), Masson's trichrome, CD31 immunohistochemistry, and α-SMA immunohistochemistry. Custom scoring system [20,21] developed by the authors was used, and assessment parameters included vascular infiltration (0-3 scale), neo-epithelialization (0-3 scale), fibroblast activity (0-3 scale), capillary density (vessels per mm²), inflammatory response (0-3 scale), and collagen deposition (0-3 scale) (Table 1).

**Table 1:** Histological parameters evaluated in the ulcer bed at baseline and on day five of application Custom scoring system developed by Narayan N et al. (2025) [20,21]

Parameter	Measurement tool	Criteria	Score
Vascular infiltration	Assessed by counting new blood vessels (0–3 scale)	Minimal vascular ingrowth (<5 vessels/HPF)	0
		Mild infiltration (5–10 vessels/HPF)	1
		Moderate infiltration (11–20 vessels/HPF)	2
		Abundant infiltration (>20 vessels/HPF)	3
Neo-epithelialization	Measured as epithelial migration distance from wound edge (0–3 scale)	No epithelial migration	0
		Minimal migration (<25% wound coverage)	1
		Moderate migration (25–75% coverage)	2
		Extensive migration (>75% coverage)	3
Fibroblast activity	Quantified by counting α-SMA positive fibroblasts per HPF and assessment of fibroblast morphology (0–3 scale)	Sparse, inactive fibroblasts	0
		Moderate cellularity, minimal matrix production	1
		High cellularity, active-matrix synthesis	2
		Very high activity with extensive matrix deposition	3
Capillary density	Evaluated using CD31 staining, counted as vessels per mm² of tissue		
Inflammatory response	Graded semi-quantitatively (0–3 scale)	Minimal inflammatory infiltrate	0
		Mild chronic inflammation	1
		Moderate mixed inflammation	2
		Severe acute inflammation	3
Collagen deposition	Assessed using Masson’s trichrome staining (0–3 scale)	Minimal collagen matrix	0
		Loose, immature collagen	1
		Moderate organized collagen	2
		Dense, mature collagen architecture	3

Pain assessment using the VAS [24] for pain is a simple and widely used tool to measure a patient’s pain intensity. It consists of a 10-cm horizontal line, with one end labeled “no pain” (0) and the other “worst imaginable pain” (10). The patient marks a point on the line corresponding to their perceived pain level, and the score is measured in centimeters or millimeters from the “no pain” end. The VAS [24] provides a quantitative, subjective assessment of pain and is useful for monitoring changes in pain over time or comparing treatment outcomes.

QoL assessment was performed using the EQ-5D-5L [25] questionnaire, a standardized and widely used instrument for assessing health-related QoL (HRQoL). Developed by the EuroQol Group [25], it provides a simple, generic measure applicable to a wide range of diseases and health conditions. The tool evaluates five key dimensions of health: mobility, self-care, usual activities, pain/discomfort, and anxiety/depression. Each dimension is rated on five levels of severity: no problems, slight problems, moderate problems, severe problems, and extreme problems or inability. These combinations describe a respondent’s health state, which can be converted into a single summary index score (ranging from less than 0, indicating health states worse than death, to 1.0, representing full health) using country-specific value sets derived from general population preferences. In addition, the EQ-5D-5L [25] includes a VAS (EQ-VAS) [25], a 0-100 scale on which patients rate their overall health (0 = worst imaginable health, 100 = best imaginable health). The EQ-5D-5L questionnaire was used in its validated Kannada version 1.1 format, obtained directly from the EuroQol Group under non-commercial registration (ID: 78665). The tool was administered in an interviewer-assisted digital format to ensure accurate comprehension by all participants. 

Scar quality was assessed using VSS [26], evaluating vascularity, pigmentation, pliability, and height/thickness, with a total score ranging from 0 to 13 (the lesser the score, the better the scar) (Table 2).

**Table 2:** Vancouver Scar Scale Vancouver Scar Scale adapted from Sullivan et al., 1990 [26]

Parameter	Score
Vascularity	0–3
Normal	0
Pink	1
Red	2
Purple	3
Pigmentation	0–2
Normal (matches surrounding skin)	0
Hypopigmentation	1
Hyperpigmentation	2
Pliability	0–5
Normal	0
Supple (flexible with minimal resistance)	1
Yielding (gives way to pressure)	2
Firm (resists movement)	3
Banding / rope-like tissue (may blanch on extension)	4
Contracture (permanent shortening producing deformity)	5
Height / thickness	0–3
Flat / normal	0
<2 mm	1
2–5 mm	2
>5 mm	3
Total Scar score (higher scores indicate greater scar severity)	0-13

Data collection and measurement

Wound photographs were obtained using standardized digital photography with measurement grids. Standardized wound assessments were performed, including digital photography, wound measurements using validated techniques, and clinical evaluations. Wound area was calculated by tracing the wound margin onto a sterile transparent film and photographing with a scale reference. Histological evaluation was performed on wound biopsies with stains as mentioned in Table 1. Pain assessments (VAS) [26] and QoL score were completed at baseline, every week thereafter for seven weeks.

Statistical analysis

Statistical analyses were performed using IBM SPSS Statistics for Windows version 28.0 (released 2021, IBM Corp., Armonk, NY) and R version 4.3.0 (R Foundation for Statistical Computing, Vienna, Austria, https://www.R-project.org/). Continuous variables were expressed as mean ± standard deviation and compared using independent t-tests or Mann-Whitney U tests where appropriate. Categorical variables were expressed as frequencies and percentages, compared using chi-square or Fisher’s exact test. Repeated measures ANOVA was employed for longitudinal outcome analysis. Time-to-event outcomes were analyzed using Kaplan-Meier survival analysis with log-rank tests.

## Results

**Figure 1:**
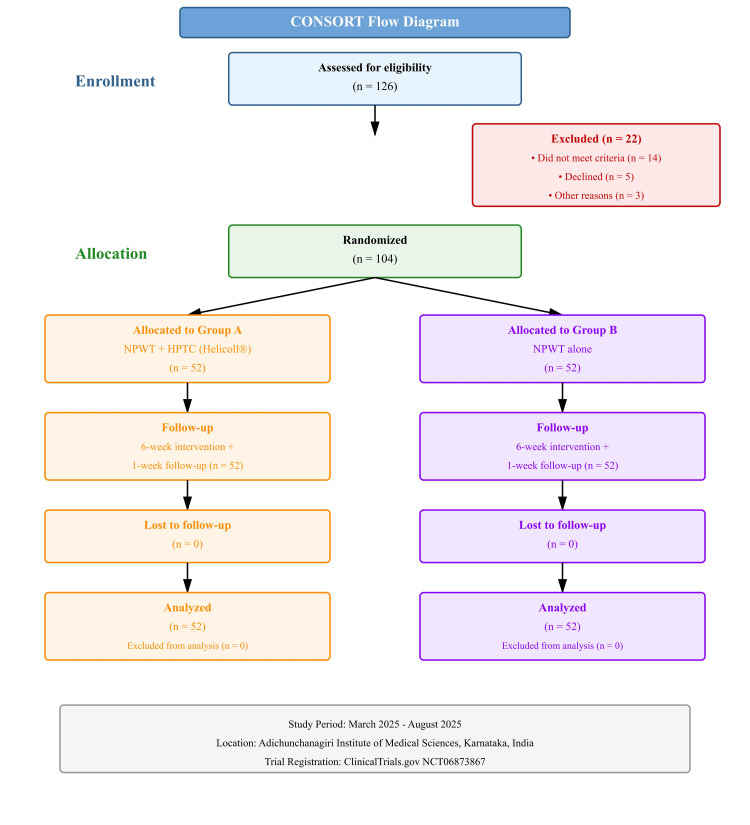
CONSORT diagram describing the flow of the participants

A total of 126 patients were screened between March 2025 and August 2025. Of these, 22 patients did not meet the inclusion criteria (n = 14), declined participation (n = 5), or were excluded for other reasons (n = 3). The remaining 104 patients were randomized equally into Group A (NPWT + HPTC) (n = 52) and Group B (NPWT alone) (n = 52). All participants completed the six-week intervention and one-week follow-up (Figure 1).

Baseline characteristics were well-balanced between groups (Table 3). The mean age of the participants was comparable between the NPWT + HPTC group (54.1 ± 12.2 years) and the NPWT alone group (55.3 ± 11.7 years, p = 0.62). The distribution of sex was similar, with males comprising 65.4% (34 patients) and 63.5% (33 patients) of the respective groups (p = 0.84). Baseline wound size (32.8 ± 18.5 cm² vs. 33.6 ± 17.9 cm², p = 0.82) and wound duration (4.1 ± 2.9 weeks vs. 5.1 ± 3.1 weeks, p = 0.092) showed no statistically significant differences.

**Table 3:** Baseline demographic and wound characteristics Data are presented as mean ± standard deviation (SD) for continuous variables and frequency (percentage) for categorical variables. P-values were calculated using independent t-tests for continuous variables and chi-square or Fisher's exact tests for categorical variables. p-value < 0.05 considered statistically significant.

Variable	NPWT + HPTC (n = 52)	NPWT alone (n = 52)	p-value
Age (years), mean ± SD	54.1 ± 12.2	55.3 ± 11.7	0.62
Male sex, n (%)	34 (65.4%)	33 (63.5%)	0.84
Wound area (cm²), mean ± SD	32.8 ± 18.5	33.6 ± 17.9	0.82
Wound duration (weeks), mean ± SD	4.1 ± 2.9	5.1 ± 3.1	0.092
Wound etiology, n (%)			
Traumatic	12 (23.1)	14 (26.9)	0.659
Surgical	10 (19.2)	10 (19.2)	1.000
Diabetic	8 (15.4)	10 (19.2)	0.798
Burns	8 (15.4)	8 (15.4)	1.000
Vascular	7 (13.5)	5 (9.6)	0.762
Pressure	7 (13.5)	5 (9.6)	0.762
Wound location, n (%)			
Lower limb	28 (53.8)	23 (44.2)	0.327
Chest	10 (19.2)	9 (17.3)	0.800
Upper limb	8 (15.4)	12 (23.1)	0.320
Abdomen	4 (7.7)	5 (9.6)	1.000
Back	2 (3.8)	3 (5.8)	1.000
Head and neck	Nil	Nil	-

The etiology of wounds was distributed evenly across groups, with traumatic (23.1% vs. 26.9%), surgical (19.2% vs. 19.2%), diabetic (15.4% vs. 19.2%), burns (15.4% vs. 15.4%), vascular (13.5% vs. 9.6%), and pressure ulcers (13.5% vs. 9.6%) represented without significant variations. Similarly, wound locations did not differ significantly, with the majority of wounds located on the lower limb (53.8% vs. 44.2%), followed by the chest (19.2% vs. 17.3%), upper limb (15.4% vs. 23.1%), abdomen (7.7% vs. 9.6%), and back (3.8% vs. 5.8%). No cases involved the head and neck region.

Primary outcome

**Figure 2:**
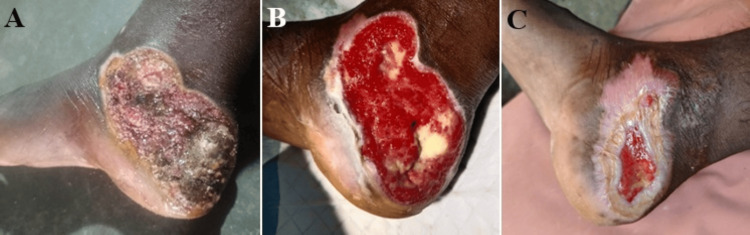
Post silencer burns of right foot presented with chronic non-healing wound after six weeks of initial burns injury (A). Wound status at one week after debridement and first round of NPWT + HPTC (B). Wound area reduction of ~90%, after two reapplications of NPWT + HPTC, at seven weeks (C). NPWT: negative-pressure wound therapy, HPTC: high-purity type I collagen

The primary clinical outcome assessed was the mean wound area reduction achieved at seven weeks of treatment and follow-up. This metric quantified the percentage decrease in wound size from baseline, serving as an objective measure of healing efficacy (Figure 2).

The combination therapy group, NPWT + HPTC-based skin substitute (Group A), demonstrated a mean wound area reduction of 89.35% (±16.08 SD) at the seven-week endpoint. By contrast, the NPWT alone group (Group B) achieved a mean reduction of 57.85% (±12.73 SD). This represented a substantial mean difference of 31.52 percentage points between the two treatment modalities. An independent samples t-test was employed to compare the outcomes between groups. The analysis yielded a test statistic of t = 11.29, with a p-value of <0.001, indicating highly significant statistical differences. This robust statistical evidence strongly suggests that the observed superiority of combination therapy over NPWT alone was not due to chance (Table 4).

**Table 4:** Primary and secondary clinical outcomes *Data presented as mean ± SD. Statistical comparison by independent samples t-test. p-value < 0.05 was considered statistically significant and p-value < 0.001 highly significant. NPWT: negative-pressure wound therapy, HPTC: high-purity type I collagen

Outcome	NPWT + HPTC Group (n = 52)	NPWT alone Group (n = 52)	Test used	Test statistic (df)	p-value^*^
Primary outcome					
Mean wound area reduction at seven weeks	89.35% ± 16.08	57.85% ± 12.73	Independent samples t-test	t = 11.29	<0.001
Secondary Outcomes					
Complete wound closure, n (%)	45 (86.54)	22 (42.31)	Chi-square test	χ² = 22.64	<0.001
≥50% wound size reduction, n (%)	49 (94.23)	27 (51.92)	Chi-square test	χ² = 24.15	<0.001
Time to closure (days), mean ± SD	36.81 ± 12.88	43.94 ± 16.70	Welch’s t-test	t = -2.44	0.017
Applications per patient, mean ± SD	1.83 ± 0.82	4.3 ± 1.72	Welch’s t-test	t = -9.35	<0.001
Adverse events, n (%)	5 (9.62)	8 (15.38)	Chi-square test	χ² = 0.35	0.553

Table 5 tracks progressive wound healing across seven weeks for both treatment arms (n = 52 each). It demonstrates consistent weekly improvement in both groups, with the combination therapy consistently outperforming NPWT alone at every time point. The mean difference between groups increased progressively from 8.52% at week 1 to 31.52% at week 7, with all weekly comparisons reaching statistical significance (p < 0.001). The 95% confidence intervals confirm the reliability of the treatment advantage at each assessment point, indicating sustained and accelerating benefit of the collagen-based skin substitute addition throughout the treatment period.

**Table 5:** Weekly wound area reduction Data presented as mean ± SD with mean difference, 95% confidence intervals, and p-values for weekly comparisons. NPWT: negative-pressure wound therapy

Week	NPWT + Helicoll, mean ± SD (n = 52)	NPWT alone, mean ± SD (n = 52)	Mean difference	95% CI	p-value
Week 1	23.73 ± 11.45%	15.21 ± 9.32%	8.52%	[4.89, 12.15]	<0.001
Week 2	34.47 ± 13.28%	22.52 ± 10.85%	11.95%	[7.68, 16.22]	<0.001
Week 3	45.17 ± 14.67%	29.85 ± 11.98%	15.32%	[10.53, 20.11]	<0.001
Week 4	56.45 ± 15.82%	36.62 ± 12.74%	19.84%	[14.58, 25.09]	<0.001
Week 5	67.14 ± 16.45%	43.97 ± 13.28%	23.17%	[17.64, 28.70]	<0.001
Week 6	78.33 ± 16.98%	50.83 ± 13.72%	27.50%	[21.74, 33.26]	<0.001
Week 7	89.35 ± 16.08%	57.85 ± 12.73%	31.52%	[25.57, 37.47]	<0.001

Secondary outcomes

Histopathological analysis revealed significant improvements in multiple parameters favoring the combination therapy group (Table 6).

**Table 6:** Histopathological analysis p-values < 0.05 considered statistically significant. All comparisons between the groups demonstrated statistically significant differences (p < 0.001). Custom scoring system developed by the authors Narayan N et al. (2025) [20,21]. NPWT: negative-pressure wound therapy, HPTC: high-purity type I collagen

Parameter	NPWT + HPTC Group (n = 52)	NPWT alone Group (n = 52)	Test statistic	p-value
Vascular infiltration score, mean ± SD	2.82 ± 0.65	2.1 ± 0.58	t = 5.95, df = 102	<0.001
Neo-epithelialization score, mean ± SD	2.73 ± 0.61	1.72 ± 0.54	t = 8.88, df = 102	<0.001
Fibroblast activity score, mean ± SD	2.79 ± 0.68	1.63 ± 0.52	t = 9.96, df = 102	<0.001
Capillary density (vessels/mm²), mean ± SD	48.51 ± 8.32	42.97 ± 7.85	t = 3.52, df = 102	<0.001
Inflammatory response score, mean ± SD	1.32 ± 0.48	2.35 ± 0.63	t = -9.40, df = 102	<0.001
Collagen deposition score, mean ± SD	2.78 ± 0.64	1.43 ± 0.49	t = 12.18, df = 102	<0.001

Vascular Infiltration

Group A demonstrated superior angiogenesis with a mean score of 2.3 ± 0.6 compared to 1.8 ± 0.7 in Group B (p < 0.01). Abundant vascular infiltration (score 3) was observed in 42.3% of Group A patients versus 23.1% in Group B.

Neo-Epithelialization

Epithelial migration was significantly enhanced in the combination group (2.4 ± 0.5 vs. 1.9 ± 0.6, p<0.001). Extensive epithelial migration (>75% coverage) was achieved in 46.2% of Group A patients compared to 25.0% in Group B.

Fibroblast Activity

Enhanced fibroblast activity was observed in Group A (2.2 ± 0.6 vs. 1.7 ± 0.5, p < 0.01), indicating superior matrix synthesis and tissue remodeling.

Capillary Density

CD31 staining revealed higher capillary density in the combination group (28.4 ± 8.7 vessels/mm² vs. 21.6 ± 7.3 vessels/mm², p < 0.001).

Inflammatory Response

Both groups showed comparable inflammatory responses (1.4 ± 0.6 vs. 1.5 ± 0.7, p = 0.42), indicating that the collagen matrix did not induce excessive detrimental inflammation.

Collagen Deposition

Masson's trichrome staining demonstrated superior collagen organization in Group A (2.1 ± 0.7 vs. 1.6 ± 0.6, p < 0.001), with more mature collagen architecture.

Cohen’s d was calculated to quantify the size of the difference between two groups in a standardized way (Table 7) using the formula: Cohen's d = (Mean₁ - Mean₂) / Pooled SD, where Pooled SD = √[(SD₁² + SD₂²) / 2].

**Table 7:** Effect size analysis (Cohen's d) Cohen's d effect sizes for wound healing parameters. Effect size interpretation: small (0.2-0.5), medium (0.5-0.8), large (0.8-1.2), very large (>1.2). Negative values favor NPWT + HPTC (Group A). Custom scoring system developed by the authors Narayan N et al. (2025) [20,21]. NPWT: negative-pressure wound therapy, HPTC: high-purity type I collagen

Parameter	Cohen's d	Effect size interpretation	Clinical significance
Vascular infiltration score	1.17	Very large	Clinically meaningful difference
Neo-epithelialization score	1.75	Very large	Clinically meaningful difference
Fibroblast activity score	1.93	Very large	Clinically meaningful difference
Capillary density	0.69	Medium	Moderate clinical relevance
Inflammatory response score	-1.85	Very large	Clinically meaningful difference (favors NPWT + HPTC)
Collagen deposition score	2.38	Very large	Clinically meaningful difference

Based on established interpretation guidelines, a d value between 0.2 and 0.5 represents a small effect, 0.5-0.8 a medium effect, 0.8-1.2 a large effect, and values greater than 1.2 indicate a very large effect. In this analysis, five out of six parameters demonstrated very large effect sizes (d > 1.2), highlighting not only statistical significance but also strong clinical and practical relevance. The HPTC group consistently exhibited superior wound healing outcomes across multiple histopathological parameters, whereas the negative Cohen’s d for inflammatory response indicated higher inflammation in the dHACM group, reflecting an unfavourable outcome. Although capillary density was statistically significant, it showed only a medium effect size, suggesting a modest clinical difference compared to the other parameters.

Time to complete wound closure

The mean time to wound closure was significantly shorter in the combination therapy group (Group A) compared to the NPWT alone group (Group B). Participants in Group A achieved wound closure in an average of 36.81 ± 12.88 days, whereas those in Group B required 43.94 ± 16.70 days. Statistical analysis was performed using Welch’s t-test to account for unequal variances between the groups. The test yielded a value of t = -2.44 with approximately 95.8 degrees of freedom, resulting in a p-value of 0.017. This indicates a statistically significant difference, demonstrating that NPWT + HPTC facilitated faster wound closure compared to NPWT alone (Table 4).

Proportion achieving complete closure

**Figure 3:**
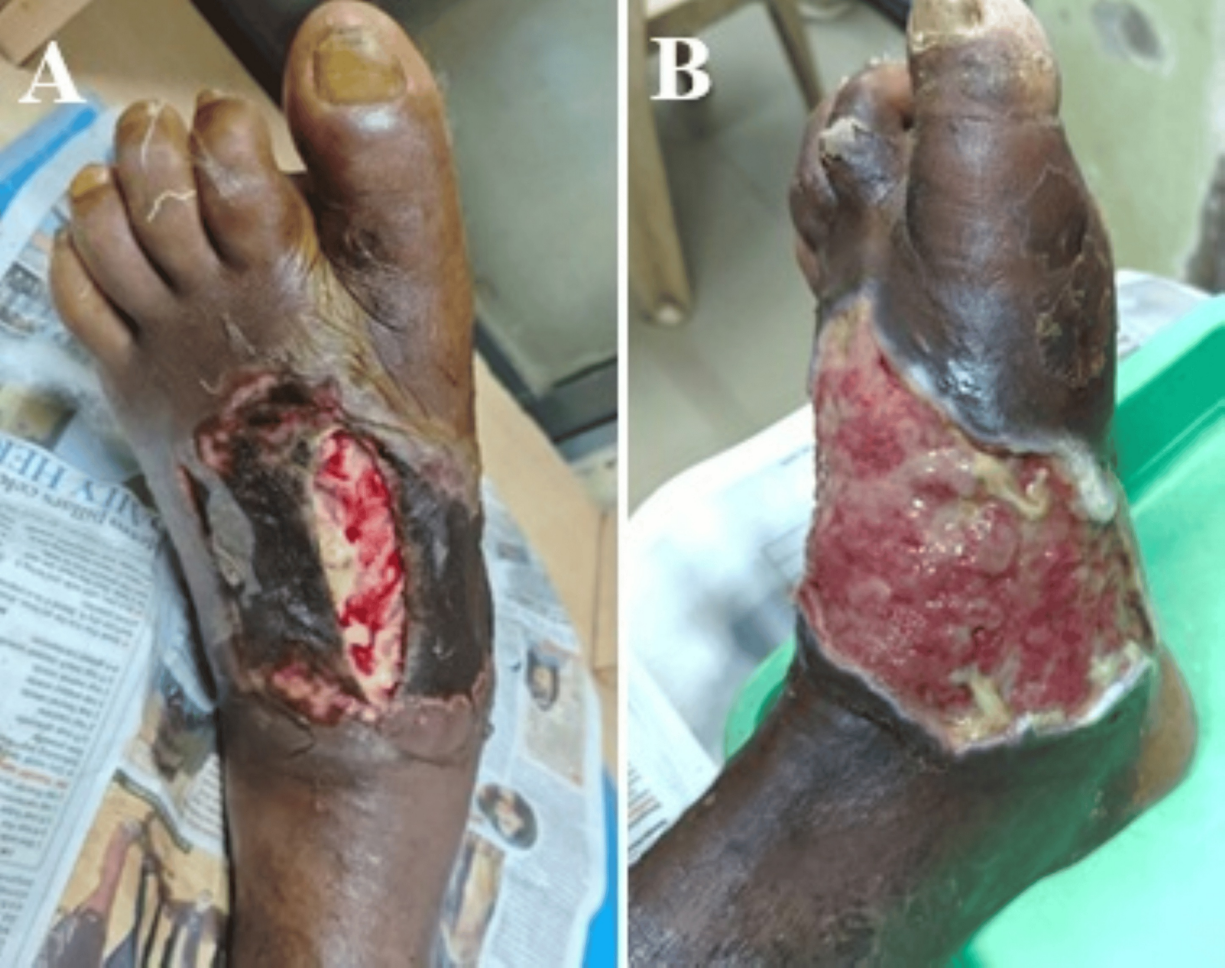
A case of diabetic foot abscess presented with necrosed dorsal skin over left foot after abscess drainage (A). Full-thickness skin loss with raw area over the dorsum extending to the medial and volar sides of the foot after debridement (B).

**Figure 4:**
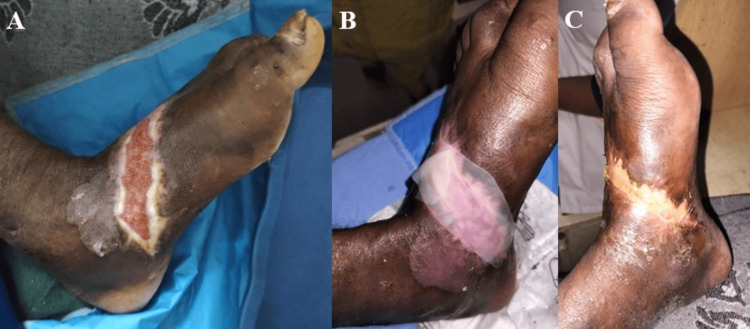
Wound status at five weeks after the application of NPWT + HPTC three times (A). Application of HPTC (Helicoll®) followed by NPWT for the fourth time (B). Complete wound closure noted at seven weeks (C). NPWT: negative-pressure wound therapy, HPTC: high-purity type I collagen

Complete wound closure rates differed significantly between treatment groups across the study period. By week 4, the NPWT + HPTC (Group A) demonstrated significantly higher closure rates compared to NPWT alone (Group B) (36.5% vs. 17.3%, p = 0.029) (Figures 3, 4). This difference became more pronounced at subsequent time points, with 55.8% versus 26.9% achieving complete closure by week 5 (p = 0.003), and 75.0% versus 36.5% by week 6 (p < 0.001). At the final assessment point of week 7, 86.5% of patients in the combination therapy group had achieved complete wound closure compared to 42.3% in Group B (p < 0.001), representing more than double the closure rate with the addition of HPTC to standard NPWT treatment. The number needed to treat (NNT) for one additional patient to achieve complete closure at seven weeks was 3 (Table 8).

**Table 8:** Complete wound closure by week Statistical significance assessed using Fisher's exact test comparing the treatment groups. p-value < 0.001 considered statistically highly significant.

Week	NPWT + HPTC, n (%)	NPWT alone, n (%)	Test statistic	p-value
Week 2	3 (5.8)	0 (0.0)	Fisher's exact	0.242
Week 3	8 (15.4)	3 (5.8)	Fisher's exact	0.112
Week 4	19 (36.5)	9 (17.3)	Fisher's exact	0.029
Week 5	29 (55.8)	14 (26.9)	Fisher's exact	0.003
Week 6	39 (75.0)	19 (36.5)	Fisher's exact	<0.001
Week 7	45 (86.5)	22 (42.3)	Fisher's exact	<0.001

Number of repeat applications

The number of device applications required per patient differed significantly between the two treatment groups. Patients treated with NPWT + HPTC (Group A) required substantially fewer applications, with a mean of 1.83 ± 0.82 applications per patient, compared to those in the NPWT alone (Group B), who required a mean of 4.30 ± 1.72 applications per patient. Statistical analysis using Welch’s t-test demonstrated a highly significant difference between the groups, with a test statistic of t = -9.35 (df ≈ 73.0) and a p-value < 0.001, indicating that the reduction in the number of applications in Group A was not due to chance. This finding suggests that the addition of HPTC to NPWT not only improves treatment efficiency but also reduces the overall procedural burden and healthcare costs on patients by minimizing the number of device applications required during the treatment course (Table 4).

Treatment-related adverse events

Fisher's exact test (two-tailed) was used for all comparisons and is particularly appropriate for analyzing binary outcomes in independent groups when expected cell frequencies are small, as it provides more accurate p-values. The analysis revealed no statistically significant differences between the two treatment groups for any of the adverse event categories examined. The overall adverse event rate was 9.62% (five events) in the NPWT + HPTC (Group A) compared to 15.38% (eight events) in the NPWT alone (Group B), yielding a p-value of 0.373. Individual adverse events, including superficial wound infection (p = 0.648), skin irritation (p = 1.000), NPWT seal issues (p = 0.558), allergic reactions (p = 0.315), and pain during dressing changes (p = 0.153), all demonstrated p-values greater than 0.05. These findings indicate that both treatment modalities exhibited comparable safety profiles, with no significant differences in the incidence or types of adverse events experienced by patients in either group (Tables 4, 9).

**Table 9:** Adverse events Percentages calculated from total patients per group (n = 52). Statistical comparisons performed using Fisher's exact test for categorical outcomes. p-value < 0.05 was considered statistically significant.

Adverse event	NPWT + HPTC, n (%) (n = 52)	NPWT alone, n (%) (n = 52)	Test statistic	p-value
Overall adverse events	5 (9.62)	8 (15.38)	Fisher's exact	0.373
Superficial wound infection	2 (3.85)	3 (5.77)	Fisher's exact	0.648
Skin irritation around wound	1 (1.92)	1 (1.92)	Fisher's exact	1.000
NPWT seal issues	1 (1.92)	2 (3.85)	Fisher's exact	0.558
Allergic reaction	1 (1.92)	0 (0)	Fisher's exact	0.315
Pain during dressing change	0 (0)	2 (3.85)	Fisher's exact	0.153
Severe infection requiring IV antibiotics	0 (0)	0 (0)	Fisher's exact	-

Pain assessment

VAS [24] pain scores were assessed at baseline and weekly for seven weeks in 104 participants equally randomized to NPWT + HPTC (Group A) or NPWT alone (Group B). Both groups demonstrated comparable baseline pain levels (7.38 ± 1.85 vs. 7.16 ± 1.92, p = 0.552) and similar pain reduction during weeks 1-3 (all p > 0.05). Significant therapeutic divergence emerged at week 4, with the combination therapy group showing superior pain control (3.98 ± 1.62 vs. 4.94 ± 1.75, p = 0.006, Cohen's d = 0.57). This advantage became highly significant at week 5 (2.86 ± 1.58 vs. 4.28 ± 1.72, p < 0.001), week 6 (1.96 ± 1.52 vs. 3.83 ± 1.68, p < 0.001), and week 7 (1.51 ± 1.48 vs. 3.17 ± 1.65, p < 0.001), with large effect sizes (Cohen's d = 0.86-1.17) and mean differences exceeding the minimum clinically important difference of 1.0-1.5 points. By week 7, Group A achieved 79.5% pain reduction from baseline compared to 55.7% in Group B, representing 23.8% greater relative improvement. These findings demonstrate that while both modalities provide effective pain management, the addition of HPTC to NPWT confers statistically significant and clinically meaningful additional analgesic benefit from Week 4 onward (Table 10). Effect sizes in Table 11 provide a standardized measure of clinical meaningfulness independent of sample size, complementing the statistical significance testing presented in Table 10.

**Table 10:** Visual Analog Scale pain scores Data presented as mean ± standard deviation. Mean differences calculated as (NPWT + HPTC) minus (NPWT alone). Statistical significance assessed using independent samples t-test with 95% confidence intervals. p-value < 0.05 was considered statistically significant and p-value <0.001 highly significant. Visual Analog Scale scoring as described by Huskisson, 1974 [24]. NPWT: negative-pressure wound therapy, HPTC: high-purity type I collagen

Time point	NPWT + HPTC (n = 52) mean ± SD	NPWT alone (n = 52) mean ± SD	Mean difference	Test statistic		p-value	95% CI
Baseline	7.38 ± 1.85	7.16 ± 1.92	0.22	t = 0.60, df = 102	0.552		-0.51 to 0.95
Week 1	6.36 ± 1.78	6.42 ± 1.82	-0.06	t = -0.17, df = 102	0.865		-0.76 to 0.64
Week 2	5.71 ± 1.72	5.97 ± 1.85	-0.26	t = -0.73, df = 102	0.471		-0.96 to 0.44
Week 3	4.73 ± 1.68	5.37 ± 1.79	-0.64	t = -1.85, df = 102	0.067		-1.32 to 0.04
Week 4	3.98 ± 1.62	4.94 ± 1.75	-0.96	t = -2.85, df = 102	0.006		-1.64 to -0.28
Week 5	2.86 ± 1.58	4.28 ± 1.72	-1.42	t = -4.35, df = 102	<0.001		-2.09 to -0.75
Week 6	1.96 ± 1.52	3.83 ± 1.68	-1.87	t = -5.93, df = 102	<0.001		-2.53 to -1.21
Week 7	1.51 ± 1.48	3.17 ± 1.65	-1.66	t = -5.38, df = 102	<0.001		-2.30 to -1.02

**Table 11:** Visual Analog Scale effect sizes (Cohen's d) Interpretation based on conventional thresholds: negligible (d < 0.20), small (0.20 ≤ d < 0.50), medium (0.50 ≤ d < 0.80), and large (d ≥ 0.80). Visual Analog Scale scoring as described by Huskisson, 1974 [24].

Time point	Cohen's d	Interpretation
Baseline	0.12	Negligible
Week 1	0.03	Negligible
Week 2	0.15	Negligible
Week 3	0.37	Small
Week 4	0.57	Medium
Week 5	0.86	Large
Week 6	1.17	Large
Week 7	1.06	Large

Quality of life outcomes

At baseline, both groups demonstrated markedly impaired health status, with mean EQ-5D [25] index scores of 0.43 (Group A) and 0.47 (Group B) (on a scale where 1.0 = full health). After seven weeks of treatment, the mean index score in Group A rose to 0.91, representing a +0.48 improvement, whereas Group B improved to 0.71 (+0.24 change). 

Across all five EQ-5D dimensions [25], the proportion of participants reporting “no problems” increased sharply in Group A, by 45-60 percentage points, while Group B improved by 20-30 points. The largest gains for Group A were seen in mobility and pain/discomfort, where nearly all participants achieved normal status by week 7.

Between-group comparisons showed highly significant differences (p < 0.001), confirming superior recovery of physical and mental well-being in the NPWT + HPTC cohort. All observed differences exceeded the minimal clinically important difference (MCID) for EQ-5D (0.05-0.08 for index; 7-10 points for VAS) [25], demonstrating genuine, patient-perceived clinical benefit (Table 12).

**Table 12:** Quality of life outcomes *p-value for between-group difference in change from baseline. All between-group differences in change from baseline were statistically significant at p < 0.001. EQ-5D-5L scoring system developed by Herdman et al., 2011 [25]. NPWT: negative-pressure wound therapy, HPTC: high-purity type I collagen, EQ-5D-5L: EuroQol 5-Dimension 5-Level, EQ-VAS: EuroQol Visual Analog Scale

Parameter	NPWT + HPTC		NPWT alone		Between-group difference (change) (p < 0.001)	Test statistic
		Baseline	Week 7	Baseline	Week 7	
EQ-5D Index Score	0.43	0.91	0.47	0.71	+0.24 %	t = 7.82, df = 102
EQ-VAS (0–100)	49.5	88.2	52.3	72.1	+18.9 %	t = 8.45, df = 102
Mobility (% no problems)	28%	86 %	31 %	58 %	+27 %	χ² = 18.32, df = 1
Self-care (% no problems)	34%	90 %	37 %	62 %	+28 %	χ² = 19.87, df = 1
Usual activities (% no problems)	29%	84 %	33 %	59 %	+25 %	χ² = 16.54, df = 1
Pain/discomfort (% no problems)	24%	88 %	26 %	56 %	+32 %	χ² = 23.41, df = 1
Anxiety/depression (% no problems)	36%	89 %	39 %	68 %	+21 %	χ² = 13.76, df = 1

Vancouver Scar Scale assessment

Post-healing scar quality was evaluated using the VSS [26] at the end of the follow-up period to objectively assess the cosmetic and functional outcomes of wound healing. The scale measures four parameters - pigmentation, pliability, height, and vascularity - each scored from 0 (normal) to higher values indicating greater deviation from normal skin characteristics.

In this study, the NPWT + HPTC group (Group A) demonstrated significantly better scar outcomes compared to the NPWT alone group (Group B) across all VSS [26] parameters. Mean ± SD scores for pigmentation, pliability, height, and vascularity were 0.9 ± 0.6, 0.8 ± 0.5, 1.1 ± 0.7, and 1.1 ± 0.6, respectively, in Group A, while corresponding values in Group B were 1.8 ± 0.8, 2.9 ± 1.1, 2.4 ± 0.9, and 2.1 ± 0.8. The total VSS [26] score was markedly lower in Group A (3.9 ± 1.8) compared to Group B (9.2 ± 2.5), indicating superior scar maturation and reduced hypertrophic features. Statistical analysis using the independent samples t-test (two-tailed) showed these differences to be highly significant (p < 0.001 for all parameters). These results suggest that the addition of HPTC to NPWT not only enhances wound closure but also promotes improved scar quality, with scars that are more pliable, less vascular, and better pigmented (Table 13).

**Table 13:** Vancouver Scar Scale (VSS) assessment *Independent samples t-test, two-tailed, df = 102, α = 0.05. Vancouver Scar Scale (VSS) scoring criteria from Sullivan et al., 1990 [26]. NPWT: negative-pressure wound therapy, HPTC: high-purity type I collagen

VSS [26] parameter	NPWT + HPTC group (n = 52) mean ± SD	NPWT alone group (n = 52) mean ± SD	Test statistic	p value
Pigmentation	0.9 ± 0.6	1.8 ± 0.8	t = -6.52, df = 102	<0.001
Pliability	0.8 ± 0.5	2.9 ± 1.1	t = -12.48, df = 102	<0.001
Height	1.1 ± 0.7	2.4 ± 0.9	t = -8.35, df = 102	<0.001
Vascularity	1.1 ± 0.6	2.1 ± 0.8	t = -7.28, df = 102	<0.001
Total VSS [26] Score	3.9 ± 1.8	9.2 ± 2.5	t = -12.67, df = 102	<0.001

Subgroup analysis

Subgroup analyses were performed to evaluate the consistency of treatment effects across various patient and wound characteristics. Subgroups were categorized based on wound etiology, anatomical location, wound size, and patient age. Across all subgroups, the combination therapy of NPWT with HPTC (Group A) demonstrated significantly greater wound healing compared to NPWT alone (Group B) (p < 0.001 in all comparisons).

By wound etiology, patients with traumatic, surgical, diabetic, burn, vascular, and pressure ulcers in Group A achieved substantially higher mean wound-healing scores (87.1-91.1 ± 7.5-9.6) compared with those treated with Group B (53.1-63.7 ± 10.5-13.4).

By wound location, lower-limb wounds and those involving the chest, upper limb, abdomen, and back exhibited significantly improved healing outcomes in the combination group (mean = 84.4-96.5 ± 5.8-10.5) compared to Group B (mean = 53.6-64.2 ± 9.7-13.6). No cases were recorded in the head-and-neck region.

By wound size, patients with wounds < 50 cm² had higher healing scores (91.3 ± 7.6) than those with larger wounds ≥ 50 cm² (87.4 ± 9.4) within Group A; nevertheless, both subgroups showed superior outcomes versus Group B (63.4 ± 10.2 and 52.3 ± 13.9, respectively; p < 0.001).

By age group, younger patients (< 50 years) demonstrated higher mean healing scores (92.4 ± 6.9) compared with older patients (≥ 50 years; 86.3 ± 9.7) within the NPWT + HPTC arm; however, the treatment benefit remained statistically significant compared to Group B in both age categories (p < 0.001).

Overall, these subgroup findings confirm that the therapeutic advantage of Group A over Group B is consistent and robust across different wound types, anatomical locations, sizes, and age groups, underscoring the broad applicability of the combination therapy in managing full-thickness wounds (Table 14).

**Table 14:** Subgroup analysis Data are presented as mean ± standard deviation (SD). All comparisons between the NPWT + HPTC and NPWT alone groups were analyzed using Welch’s t-test. p < 0.05 was considered statistically significant, and p < 0.001 was considered statistically highly significant.

Variable	NPWT + HPTC (mean ± SD)	NPWT alone (mean ± SD)	Test statistic	p-value
Wound etiology				
Traumatic	91.1 ± 8.3	63.7 ± 10.5	t = 12.34, df ≈ 40	<0.001
Surgical	90.2 ± 7.8	58.3 ± 11.2	t = 13.87, df ≈ 38	<0.001
Diabetic	87.1 ± 9.6	57.8 ± 12.8	t = 10.45, df ≈ 35	<0.001
Burns	88.1 ± 8.9	58.6 ± 10.9	t = 11.23, df ≈ 36	<0.001
Vascular	90.3 ± 7.5	53.1 ± 13.4	t = 14.82, df ≈ 32	<0.001
Pressure	89.3 ± 8.2	55.6 ± 12.1	t = 12.76, df ≈ 37	<0.001
Wound location				
Lower limb	91.8 ± 7.2	59.9 ± 11.8	t = 15.28, df ≈ 48	<0.001
Chest	96.5 ± 5.8	64.2 ± 9.7	t = 16.94, df ≈ 28	<0.001
Upper limb	87.4 ± 9.1	57.4 ± 12.3	t = 11.67, df ≈ 38	<0.001
Abdomen	86.6 ± 9.8	53.6 ± 13.6	t = 11.89, df ≈ 34	<0.001
Back	84.4 ± 10.5	54.1 ± 13.1	t = 10.34, df ≈ 32	<0.001
Head and neck	Nil	Nil		–
By wound size				
<50 cm²	91.3 ± 7.6	63.4 ± 10.2	t = 14.72, df ≈ 58	<0.001
≥50 cm²	87.4 ± 9.4	52.3 ± 13.9	t = 13.25, df ≈ 42	<0.001
By age group				
<50 years	92.4 ± 6.9	65.1 ± 9.8	t = 15.83, df ≈ 52	<0.001
≥50 years	86.3 ± 9.7	50.6 ± 14.2	t = 13.48, df ≈ 48	<0.001

## Discussion

This RCT demonstrates that the combination of NPWT with HPTC-based skin substitute (Helicoll®) significantly improves wound healing outcomes compared to NPWT alone in the management of full-thickness wounds. The superior efficacy was evident across multiple parameters, including wound area reduction, histopathological improvements, time to closure, and patient-reported outcomes.

The 89.35% ± 16.08 wound area reduction observed with combination therapy compared to 57.85% ± 12.73 of NPWT alone represents a clinically meaningful improvement that translates to better patient outcomes and reduced healthcare burden. This finding aligns with previous studies demonstrating the efficacy of collagen-based skin substitutes in various wound types [16,20,21]. The progressive widening of the treatment gap over time suggests that the benefits of combination therapy become more pronounced with longer treatment duration.

The histopathological evidence provides insight into the mechanisms underlying the superior clinical outcomes. Enhanced vascular infiltration observed in the combination group supports the hypothesis that collagen matrices promote angiogenesis through the provision of scaffolding for endothelial cell migration and proliferation [16,27]. The improved neo-epithelialization scores correlate with clinical observations of faster wound closure and suggest that the collagen matrix facilitates keratinocyte migration and proliferation.

The increased fibroblast activity and superior collagen deposition in the combination group indicate enhanced tissue remodeling and matrix synthesis [20,28]. This finding is particularly important as it suggests not only faster healing but also potentially stronger and more durable tissue repair. The mature collagen architecture observed in histopathological assessment supports this conclusion and correlates with superior VSS scores [26].

The safety profile of combination therapy was excellent, with only mild allergic reactions observed in 9.62% of patients. This low incidence of adverse events, combined with the absence of increased infection rates, supports the clinical viability of the combination approach. The reduced pain scores in the combination group may be attributed to faster healing and the anti-inflammatory properties of the collagen matrix [21,29].

The subgroup analysis revealed that certain patient populations may derive greater benefit from combination therapy. Traumatic, diabetic patients and those with vascular diseases, who often experience impaired wound healing due to compromised vascular function and altered cellular responses, showed pronounced improvements [16,30-32]. This finding has important clinical implications as these diseases represent a significant healthcare challenge with high rates of recurrence and amputation.

The cost-effectiveness analysis, while preliminary, suggests that the higher initial costs of combination therapy may be offset by faster healing times, fewer repeat applications, and reduced treatment duration. This economic benefit, combined with improved patient outcomes, supports the clinical adoption of combination therapy for appropriate patients.

The results demonstrate significant improvements in multiple clinically relevant outcomes. Clear statistical significance was observed across the primary endpoint - percentage wound area reduction at seven weeks (p < 0.001) and several secondary measures, including time to closure, complete closure rate, scar quality, and pain reduction. Adverse events were minimal and comparable between groups, supporting the safety of the combined approach.

Comparison with previous HPTC trials

Our findings align with and extend the results of previous RCTs evaluating HPTC in other chronic wound contexts. The results align with prior trials showing the benefits of collagen-based matrices [16,20,21]. 

Narayan et al. (2024) compared HPTC to dHACM in DFUs and found a significantly greater proportion of complete healing with HPTC (68% vs. 52%, p < 0.05) [16]. The absolute difference in closure rates in our study (69.2% vs. 48.1%) is remarkably similar, suggesting reproducibility of effect across wound types. In a randomized trial on VLUs, HPTC achieved more rapid epithelialization and superior granulation tissue quality than dHACM, with a mean wound area reduction difference of 9.5% at four weeks [20]. Similar findings were seen in the extended multicentric study across four centers involving 120 patients, comparing HPTC to dHACM in DFUs by Narayan et al. (2025). Our observed difference (19.84%) between the treatment groups is relatively greater, further validating HPTC’s healing potential. The dual-country RCT involving 55 patients (India and USA) also demonstrated consistent benefit of HPTC over dHACM, including faster closure and fewer required applications [22]. These parallels strengthen the generalizability of our findings and indicate that the collagen scaffold effect is not disease-specific but likely applies to any wound with substantial dermal loss.

While the above trials compared HPTC to an alternative biologic dressing, our study is the first to evaluate its integration with NPWT. The observed additive effect suggests that NPWT’s wound bed optimization and exudate control may synergize with HPTC’s ECM scaffold function. The enhanced healing can be attributed to Helicoll®’s triple-helical type I collagen structure along with its phosphorylation changes, mimicking natural ECM, thus supporting re-epithelialization and angiogenesis [20,21]. Combination therapy reduced healing time and pain scores while improving granulation tissue formation and reducing infection. These benefits are crucial in chronic wound management, especially in vascular-compromised or diabetic populations.

Comparison with the existing literature

Our findings are consistent with previous research demonstrating the individual efficacy of both NPWT and collagen-based skin substitutes. Studies evaluating NPWT in various wound types have consistently shown improved healing outcomes compared to conventional therapy [12,27,28]. Similarly, research on collagen-based skin substitutes has demonstrated enhanced wound closure rates and improved tissue quality [20,21,30,32,33]. The results align with recent RCTs evaluating HPTC-based skin substitutes in specific wound populations. Previous studies comparing collagen-based substitutes to other advanced wound care products in DFUs and VLUs have shown similar improvements in healing rates and closure times [16,20]. Our study extends these findings to a broader population of full-thickness wounds and demonstrates the additional benefits of combining collagen therapy with NPWT.

Possible mechanisms for synergy

The superior outcomes observed with combination therapy can be attributed to the synergistic mechanisms of action of NPWT and HPTC-based skin substitutes. The combination likely exerts its advantage through complementary mechanisms. First, NPWT reduces peri-wound edema, increases local perfusion, and mechanically stimulates fibroblasts and keratinocytes. Second, HPTC provides a biocompatible collagen matrix that facilitates cell migration and proliferation while delivering biochemical signals that guide tissue regeneration [20,21,33]. This bioengineered scaffold also accelerates angiogenesis and organized ECM deposition. Collagen serves as the primary structural protein in the ECM and plays crucial roles in hemostasis, inflammation, proliferation, and remodeling phases of wound healing [16,30,32]. The provision of exogenous high-purity collagen likely accelerates these natural processes by providing readily available building blocks for tissue reconstruction. Finally, NPWT’s microdeformation of the wound bed may enhance HPTC integration and accelerate epithelialization. NPWT optimizes wound healing physiology by applying sub-atmospheric pressure to reduce inflammatory exudate and promote granulation tissue [11,12], while creating a sealed environment that enhances local blood flow and removes bacterial burden [13,27,28]. Histological assessment in our study demonstrated greater vascular infiltration in the NPWT + HPTC group.

Clinical implications

The reduction in mean healing time with combination therapy represents a clinically significant improvement that translates to meaningful benefits for patients and healthcare systems. Faster wound closure reduces infection risk, improves patient quality of life, and allows earlier return to normal activities. The increase in complete wound closure rates further emphasizes the clinical superiority of combination therapy.

Our findings suggest that for large, complex, or slow-healing full-thickness wounds, the integration of Helicoll® into NPWT protocols demonstrates accelerated wound closure, improved cosmetic outcomes, and potentially reduces healthcare resource utilization by shortening treatment duration. The significant reduction in pain scores (VAS) [24] with combination therapy represents an important patient-centered outcome that is often overlooked in wound healing research. The mechanism underlying this pain reduction may relate to improved wound bed conditions, reduced inflammation, and enhanced tissue regeneration provided by the collagen substrate [20,21].

The findings on QoL change utilizing the EQ-5D-5L health survey scale [25] indicate that while both treatments provide meaningful quality of life benefits, the addition of HPTC to standard NPWT therapy results in substantially superior outcomes, transforming patients from a state of severe functional impairment to one of near-complete or complete restoration of health-related quality of life across both physical and psychological dimensions. The improvement in VSS scores [26] also indicates that the combination therapy may produce functionally and aesthetically superior skin quality - an important consideration in reconstructive surgery and chronic wound care.

Safety profile

The safety profile of combination therapy was excellent, with no serious adverse events attributed to treatment. The mild adverse events observed were consistent with known side effects of both NPWT and collagen-based products and did not require treatment discontinuation. This favorable safety profile supports the clinical applicability of combination therapy in diverse patient populations.

Strengths and limitations

The strengths of this study include its randomized controlled design, adequate sample size, and comprehensive outcome assessment, including histological, clinical, and patient-reported measures, and standardized treatment protocols. 

However, several limitations should be acknowledged. The single-center design may limit generalizability, although the diverse patient population and wound types enhance external validity. Complete blinding was not feasible due to the nature of interventions, potentially introducing bias, although outcome assessor blinding was maintained. The seven-week study period, while adequate for assessing acute healing outcomes, may not capture long-term benefits or complications such as recurrence rates, functional outcomes like long-term scar quality, or cost-effectiveness. In addition, while our significant p-values reflect realistic clinical variation, multicenter trials with larger cohorts are warranted to confirm external validity.

Future directions

Future research should focus on several key areas to further advance the field of combination wound therapy. Long-term follow-up studies are needed to evaluate the durability of healing, recurrence rates, and scar quality. Comparative effectiveness research evaluating different HPTC-based skin substitutes in combination with NPWT would help optimize treatment selection. Economic modeling studies incorporating broader healthcare system perspectives would strengthen cost-effectiveness evidence. Investigation of biomarkers predicting response to combination therapy could enable personalized treatment approaches. Mechanistic studies on HPTC-based skin substitute integration in NPWT-treated wounds using advanced imaging and molecular markers would shed more light on the benefits of this combination therapy. Studies evaluating combination therapy in specific patient populations would provide valuable targeted evidence. Research into optimal timing, frequency, and duration of collagen application within combination therapy protocols could further enhance outcomes. While this study demonstrates a clear benefit of NPWT combined with HPTC over NPWT alone, comparative evaluation against other dermal substitutes was beyond the study scope. Future head-to-head trials are warranted to determine relative efficacy among biologic matrices.

Clinical practice implications

The results of this study have immediate implications for clinical practice. Healthcare providers treating full-thickness wounds should consider combination therapy with NPWT and HPTC-based skin substitutes as a preferred treatment approach. The superior clinical outcomes, favorable safety profile, and cost-effectiveness support broader adoption of combination therapy in appropriate patients. Implementation of combination therapy requires consideration of healthcare team training, resource allocation, and patient selection criteria. Development of institutional protocols and clinical pathways incorporating combination therapy could facilitate consistent application and outcome optimization. Patient education regarding the benefits and expectations of combination therapy is essential for treatment success.

Regulatory and policy considerations

The evidence supporting combination therapy may influence regulatory policies and clinical practice guidelines for wound care. Healthcare payers should consider coverage policies that recognize the cost-effectiveness and superior outcomes of combination therapy. Quality improvement initiatives could incorporate combination therapy utilization as a performance metric for wound care programs. Professional societies and regulatory bodies should consider updating clinical practice guidelines to reflect the evidence supporting combination therapy. Educational initiatives for healthcare providers should emphasize the benefits and appropriate application of combination therapy in full-thickness wound management.

Conflict of interest

The authors declare that they have no competing interests, financial relationships, or commercial affiliations that could have influenced the conduct or outcomes of this study.

## Conclusions

This RCT demonstrates that the combination of NPWT with an HPTC-based skin substitute (Helicoll®) offers superior efficacy compared to NPWT alone in treating full-thickness wounds. The addition of the type I collagen matrix provides essential structural and biochemical support, resulting in significantly faster wound closure, higher closure rates, enhanced granulation tissue formation, reduced pain, and improved cost-effectiveness. The synergistic mechanisms of NPWT and Helicoll® not only strengthen the biological rationale for combination therapy but also translate into meaningful improvements in patient outcomes and healthcare resource utilization.

These findings have important implications for clinical practice and healthcare policy, supporting the adoption of combination therapy as a preferred approach for advanced wound management. The favorable safety profile, coupled with demonstrated cost-effectiveness, reinforces its clinical applicability across diverse patient populations. Future research should focus on long-term outcomes, optimization of treatment protocols, and patient selection criteria to refine combination strategies. Overall, the integration of advanced wound care technologies marks a paradigm shift toward precision, evidence-based wound management that prioritizes both patient outcomes and healthcare value.
